# Genetic Variations *miR-10a*A>T, *miR-30c*A>G, *miR-181a*T>C, and *miR-499b*A>G and the Risk of Recurrent Pregnancy Loss in Korean Women

**DOI:** 10.3390/biomedicines10102395

**Published:** 2022-09-25

**Authors:** Hui-Jeong An, Sung-Hwan Cho, Han-Sung Park, Ji-Hyang Kim, Young-Ran Kim, Woo-Sik Lee, Jung-Ryeol Lee, Seong-Soo Joo, Eun-Hee Ahn, Nam-Keun Kim

**Affiliations:** 1 Department of Biomedical Science, College of Life Science, CHA University, Seongnam 13488, Korea; 2 College of Life Science, Gangneung-Wonju National University, Gangneung 25457, Korea; 3 Department of Otolaryngology-Head and Neck Surgery, College of Medicine, Soonchunhyang University Cheonan Hospital, Cheonan 31151, Korea; 4 Department of Obstetrics and Gynecology, CHA Bundang Medical Center, School of Medicine, CHA University, Seongnam 13488, Korea; 5 Fertility Center of CHA Gangnam Medical Center, CHA University, Seoul 06135, Korea; 6 Department of Obstetrics and Gynecology, Seoul National University Bundang Hospital, Seongnam 13620, Korea

**Keywords:** recurrent pregnancy loss, single-nucleotide polymorphism (SNP), microRNA

## Abstract

This study investigated the genetic association between recurrent pregnancy loss (RPL) and microRNA (miRNA) polymorphisms in *miR-10a*A>T, *miR-30c*A>G, *miR-181a*T>C, and *miR-499b*A>G in Korean women. Blood samples were collected from 381 RPL patients and 281 control participants, and genotyping of *miR-10a*A>T, *miR-30c*A>G, *miR-181a*T>C, and *miR-499b*A>G was carried out by TaqMan miRNA RT-Real Time polymerase chain reaction (PCR). Four polymorphisms were identified, including *miR-10a*A>T, *miR-30c*A>G, *miR-181a*T>C, and *miR-499b*A>G. *MiR-10a dominant model* (AA vs. AT + TT) and *miR-499b*GG genotypes were associated with increased RPL risk (adjusted odds ratio [AOR] = 1.520, 95% confidence interval [CI] = 1.038–2.227, *p =* 0.032; AOR = 2.956, 95% CI = 1.168–7.482, *p =* 0.022, respectively). Additionally, both *miR-499* dominant (AA vs. AG + GG) and recessive (AA + AG vs. GG) models were significantly associated with increased RPL risk (AOR = 1.465, 95% CI = 1.062–2.020, *p =* 0.020; AOR = 2.677, 95% CI = 1.066–6.725, *p =* 0.036, respectively). We further propose that *miR-10a*A>T, *miR-30c*A>G, and *miR-499b*A>G polymorphisms effects could contribute to RPL and should be considered during RPL patient evaluation.

## 1. Introduction

Recurrent pregnancy loss (RPL) is generally defined as three or more consecutive losses of pregnancy before 20 weeks of gestation. However, the American Society for Reproductive Medicine recently redefined RPL as more than two consecutive pregnancy losses [1]. Worldwide, RPL is a serious health problem that is significantly associated with morbidity and mortality. Factors contributing to the etiology of RPL include advanced maternal age, maternal anatomic anomalies, placental anomalies, chromosomal abnormalities, endocrine dysfunction, antiphospholipid syndrome, hereditary thrombophilia, psychological trauma, and environmental factors, such as smoking, excessive alcohol consumption, and stress [2]. Additionally, women who miscarry during their first pregnancy are 5% more likely to develop RPL than healthy women [3]. Although many relevant factors have been identified, the root cause of most cases of RPL remains unknown. RPL is also associated with blood clotting angiogenesis and immune disorders.

MicroRNAs (miRNAs) are small (approximately 23 nucleotides), noncoding, single-stranded RNA molecules that form base pairs with complementary target messenger RNAs (mRNAs) [4]. It has been demonstrated that miRNAs modulate gene expression via destabilization or translational repression of target mRNAs [5,6]. Furthermore, miRNAs have been implicated in the regulation of several biochemical pathways in various eukaryotic organisms [7,8]. RNA polymerase II transcribes miRNAs into long precursor transcripts known as primary (pri)-miRNAs, which are subsequently converted into pre-miRNAs by DROSHA, which is a ribonuclease type III enzyme that forms a functional complex with DiGeorge syndrome critical region 8 [9,10]. The pre-miRNA is then exported to the cytoplasm by the exportin5 (XPO5)-RAS–related nuclear protein (RAN)-guanosine-5′-triphosphate (GTP) complex [11]. RAN is a small GTP-binding protein, and the RAN GTPase-XPO5 complex forms a heterotrimer with the pre-miRNA [12]. The pre-miRNA is processed by RNase III DICER to release the miRNA duplex, which is a double-stranded RNA approximately 23 nucleotides in length. DICER also initiates the formation of the RNA-induced silencing complex (RISC) [13], which is responsible for miRNA-mediated gene silencing and RNA interference. The biological function of the miRNA is initiated by binding to the 3′-untranslated region (UTR) of the target mRNA, thereby repressing its expression. A single miRNA can regulate the expression of multiple target mRNAs, thus serving as a master controller of gene expression.

Multiple studies have recently demonstrated the roles of miRNAs in the pathophysiology of several ovarian diseases, including polycystic ovary syndrome (PCOS) and primary ovarian insufficiency (POI) [14,15]. POI, which is also known as premature ovarian failure, is characterized by insufficient or premature depletion of ovarian reserves, which leads to infertility [16]. The findings of the present study suggest that miRNAs play an essential role in the normal function and regulation of reproductive organs.

The expression of a given gene may be affected or regulated by its genetic variations, and single-nucleotide polymorphisms (SNPs) are the most common genetic variation affecting DNA [17]. SNPs or mutations in genes encoding miRNAs can affect miRNA properties, resulting in their altered expression and/or maturation [18]. Sequence variations around the processing sites of miRNAs or in the mature miRNA itself, particularly in the seed sequence, can profoundly affect miRNA biogenesis and function [19]. Polymorphisms in pre-miRNAs were first reported in 2005 [20], and several studies on the associations of these polymorphisms have since been reported [21,22]. Aberrant miRNA expression has been implicated in numerous diseases; therefore, considerable research efforts are currently being made for miRNA-based therapies [23]. In the present study, we performed a database search and identified four SNPs in pre-form miRNAs: *miR-10a*A>T (rs3809783), *miR-30c*A>G (rs113749278), *miR-181a*T>C (rs16927589), and *miR-499b*A>G (rs3746444). All of these miRNAs are reportedly associated with various reproductive diseases [24,25,26,27]. Therefore, we hypothesized that the SNPs *miR-10a*, *miR-30c*, *miR-181a*, and *miR-499b* play a role in the development of RPL. The minor allele frequency of these SNPs is >5% in the Asian population; however, whether they are genetically associated with RPL or whether miRNA expression varies as a function of these pre-form polymorphisms remains unclear. We, therefore, investigated the correlation between RPL and these miRNA polymorphisms.

## 2. Materials and Methods

### 2.1. Study Participants

Blood samples were collected from 381 RPL patients (mean age ± standard deviation [SD], 33.00 ± 5.73 years) and 281 control participants (33.03 ± 4.36 years). Blood samples were collected prior to 20 weeks of gestation based on human chorionic gonadotropin (hCG) levels. The RPL patients were recruited from the Department of Obstetrics and Gynecology or the Fertility Center at the CHA Bundang Medical Center in Seongnam, South Korea between March 1999 and February 2010. Women in the control group were recruited from CHA Bundang Hospital and met the following criteria: history of at least one spontaneous pregnancy; current pregnancy; regular menstrual cycles; karyotype 46, XX; and no history of miscarriage. The study abided by the Declaration of Helsinki and was approved by the Institutional Review Board of CHA Bundang Medical Center (IRB approval no. BD2010-123D), and written informed consent was obtained from all participants. All RPL patients had suffered a minimum of two consecutive spontaneous miscarriages at an average gestational stage of 7.36 ± 1.93 weeks. Pregnancy loss was diagnosed based on the results of hCG tests, ultrasound, and/or physical examination before 20 weeks of gestation. None of the participants had a history of smoking or alcohol use. The following parameters were also measured: activated partial thromboplastin time (aPTT), body mass index (BMI), blood urea nitrogen (BUN), creatinine, estradiol (E2), follicle-stimulating hormone (FSH), luteinizing hormone (LH), platelet (PLT) count, and prothrombin time (PT), using participant blood samples.

Patients with the following conditions were excluded from the study: RPL or implantation failure due to hormonal, genetic, anatomic, infectious, autoimmune, or thrombotic causes. Anatomic causes were evaluated using hysterosalpingogram, hysteroscopy, computed tomography, and magnetic resonance imaging to detect intrauterine adhesions, septate uterus, and uterine fibroids. Hormonal causes, including hyperprolactinemia, luteal insufficiency, and thyroid disease, were evaluated by blood analyses. Infectious causes, such as the presence of Ureaplasma urealyticum or Mycoplasma hominis, were evaluated by bacterial culture. Autoimmune causes, including antiphospholipid syndrome or lupus, were evaluated using lupus anticoagulant and anticardiolipin antibodies. Thrombotic causes, such as thrombophilia, were evaluated by identification of protein C and S deficiencies and by detection of β-2-glycoprotein 1 antibodies.

### 2.2. Antibody Preparation

A total of 150 μL of whole blood and fluorochrome-labeled monoclonal antibodies against anti-CD3-FITC (1:100, 555339), anti-CD4-PE(1:100, 357404) anti-CD8-PE-cy5 (1:20, 344769) anti-CD19-APC (1:100, 392503), anti-CD56-PE-Cy7 (1;100, 392411) NK cells were added to each tube. All antibodies were obtained from Biolegend (San Diego, CA, USA). The tubes were vortexed and incubated in the dark at room temperature for 40 min. Next, 2 mL of Lyse solution (diluted 1:10; BD Bioscience, Sunnyvale, CA, USA) was added, and the tubes were vortexed again, incubated at room temperature for 30 min, and centrifuged at 1200 rpm for 5 min. The cells were then washed three times with 2 mL of PBS each wash, and the cells were suspended in 250 μL of PBS and analyzed by flow cytometry (BD Bioscience).

### 2.3. Chromosome Analysis

 Chromosome analysis was conducted according to standard cytogenetic methods. Peripheral blood lymphocytes were cultured for 70 h, and then KaryoMAX Colcemid Solution (Gibco) was added when the chromosomes were at the metaphase stage. KCl (0.05 M) was added as a hypotonic agent, and the cells were fixed for harvest using a fixative formed by adding one volume of acetic acid to two volumes of methanol. Metaphase chromosome preparations obtained after cell culture were stained using the Giemsa-Trypsin-Giemsa (GTG) banding method.

### 2.4. Genotyping

Genomic DNA was extracted from anticoagulant-treated peripheral blood samples using a G-DEX Genomic DNA extraction kit (iNtRON Biotechnology, Seongnam, Korea) [28,29]. Briefly, Proteinase K was added to a microcentrifuge tube, followed by 30 µL of blood. Next, 300 µL of Lysis solution was added, and the samples were vortexed and incubated at 55 °C for 10 min. A total of 350 µL of ethanol was then added to each sample, and the samples were bound, washed, and eluted according to the manufacturer’s protocol. Four miRNAs (SNPs) were selected using the NCBI human genome SNP database (dbSNP, http://www.ncbi.nlm.nih.gov/snp (accessed on 13 March 2019)). The SNPs *miR-10a*A>T (rs3809783), *miR-30c*A>G (rs113749278), *miR-181a*T>C (rs16927589), and *miR-499b*A>G (rs37464444) are either mature-form (rs3746444, rs-formnp8978) or pri-form (rs3809783, rs16927589). *miR-10a*A>T, *miR-30c*A>G, *miR-181a*T>C, and *miR-499b*A>G were genotyped according to TaqMan^®^ SNP Genotyping Assays system (Applied Biosystems, Foster City, CA, USA). Based on the intensity of fluorescence signals of FAM and VIC, samples were automatically classified into one of three groups corresponding to the genotypes AA, AG, or TT of *miR-10a*A>T; AA, AG, or GG of *miR-30c*A>G; TT, TC, or CC of *miR-181a*T>C; and AA, AG, or GG of *miR-499b*A>G. The basic principle of the assay is as follows: when the allele-specific probe is fully hybridized to the template DNA, Taq polymerase cleaves the reporter dye, leading to fluorescence emission. However, if a single base mismatch exists between the probe and template DNA, hybridization is inefficient, and reporter dye fluorescence is thus reduced. The sequences of the SNPs were as follows: *miR-10a*A>T, CTCTT ATTTTTCCAG AAGAAAAAAA[A/T]ATATATATAT GTATATGTAG TATTT; *miR-30c*A>G, TACTTTCCACAGCTG AGAGTGTAGG[A/G]DTGTTTACAGT ATCTGTCGCT CAGTG; *miR-181a*T>C, AAAAT AGCACAAAAT TATCCAATTG[T/C] GACAGTTCTT ATCACATTTC ACTTT; and *miR-499b*A>G, ATGTTTAACT CCTCTCCACG TGAAC[A/G]TCACAGCAAG TCTGTGCTGC TTCCC. Information regarding the miRNA probes was as follows: *miR-10a*A>T, wild type homozygous AA (VIC reaction & FAM no reaction), heterozygous AT (VIC reaction & FAM reaction), mutant homozygous TT (VIC no reaction & FAM reaction); *miR-30c*A>G, wild homozygous AA (VIC reaction & FAM no reaction), heterozygous AG (VIC reaction & FAM reaction), mutant homozygous GG (VIC no reaction & FAM reaction); *miR-181a*T>C, wild homozygous TT (VIC reaction & FAM no reaction), heterozygous TC (VIC reaction & FAM reaction), mutant homozygous CC (VIC no reaction & FAM reaction); *miR-499b*A>G, wild homozygous AA (VIC reaction & FAM no reaction), heterozygous AG (VIC reaction & FAM reaction), mutant homozygous GG (VIC no reaction & FAM reaction).

### 2.5. Assessment of Plasminogen Activator Inhibitor (PAI-1), Homocysteine, Total Cholesterol, Uric Acid Levels, and Blood Coagulation Status

Plasma PAI-1, total cholesterol, uric acid, and homocysteine levels were measured in participant blood samples. Plasma was separated by centrifugation of whole blood at 1000× *g* for 15 min. PAI-1 levels were determined using a human serpin E1/PAI-1 immunoassay (R&D Systems, Minneapolis, MN, USA). Uric acid and total cholesterol levels were measured using enzymatic colorimetric tests (Roche Diagnostics, GmbH, Mannheim, Germany). Homocysteine levels were measured using a fluorescence polarization immunoassay with an Abbott IMx analyzer (Abbott Laboratories, Abbott Park, IL, USA).

### 2.6. Statistical Analyses

The significance of differences in the frequencies of the *miR-10a*A>T, *miR-30c*A>G, *miR-181a*T>C, and *miR-499b*A>G SNPs between the control and patient groups were assessed using Fisher’s exact test and a logistic regression model. *p*-values were calculated using two-sided *t*-tests for continuous variables and chi-square tests for categorical variables. Allele frequencies were calculated to investigate the deviation from Hardy–Weinberg equilibrium. The genotype distribution of RPL patients and controls with ≥h or ≥o pregnancy loss was investigated. Odds ratios (ORs), adjusted odds ratios (AORs), and 95% confidence intervals (CIs) were used to examine the associations between various miRNA polymorphisms and RPL risk. Data are presented as the mean ± SD for continuous variables or a percentage for categorical variables. The results of the allele and genotype combination analysis were consistent with those derived from Fisher’s exact test during regression analysis.

Statistical analyses were carried out using MedCalc software, version 12.1.4 (MedCalc Software bvba, Mariakerke, Belgium) or GraphPad Prism 4.0 software (GraphPad Software, Inc., San Diego, CA, USA). Logistic regression analysis was applied to data regarding baseline characteristics, genotype frequencies, genotype combinations, and allele combinations for quantitative traits shown in Table 2, Table 3, Table 4 and Table 5. The HAPSTAT program (v.3.0, www.bios.unc.edu/~lin/hapstat/ (accessed on 10 April 2018)), which exhibits a strong synergistic effect, was used to estimate the frequencies of polymorphic haplotypes. A *p*-value < 0.05 indicated statistical significance. HAPSTAT allows testing of haplotype (or allele combination) effects by maximizing the likelihood (from the observed data) that properly accounts for phase uncertainty and study design. False-positive discovery rate (FDR) correction was used to adjust multiple comparison tests and associations with FDR-adjusted *p*-values < 0.05 were considered statistically significant [30]. FDR calculation is also used for multiple hypotheses testing to correct for multiple comparisons. Multifactor dimensionality reduction (MDR) analysis was used to determine the best-model gene-gene interaction for RPL risk. The advantage of using MDR is that it overcomes the sample size limitations often encountered during logistic regression analysis in studies of high-level interactions. The MDR method consists of two main steps. First, the best combination of multi-factors is selected, and second, genotype combinations are classified into high- and low-risk groups [31]. We constructed all possible allelic combinations by MDR analysis to identify combinations with strong synergy. Allelic combinations for multiple loci were estimated using the expectation-maximization algorithm with SNPAlyze (v. 5.1; DYNACOM Co, Ltd., Yokohama, Japan), and those having frequencies < 1% were excluded from statistical analysis. We also applied multiple regression models to further explain the results of the allelic combination analysis. Genetic interaction analyses were performed using the open-source MDR software package (v.2.0), which is available at www.epistasis.org (accessed on 15 March 2018).

### 2.7. Expression Vector Construction (miR-10aA>T, miR-30cA>G, and miR-181aT>C)

The pre-miRs *(miR-10a*, *miR-30c*, and *miR-181a*) and their flanking regions were amplified from human genomic DNA and cloned into the vector pcDNA3.1(−) (Invitrogen, Carlsbad, CA, USA). The primers used in the study included F: 5′-TGC GAA CTG GCT ACT TGA AA-3′, R: 5′-TTC CAA TAA AGC CTC CCT GA-3′ (*miR-10a*); F: 5′-GCA CCA TGT GTC ACA CAG GT-3′, R: 5′-CAA GTG TTG GGA AGA TGC TAT-3′ (*miR-30c*); and F: 5′-ACA TTT TCT CAG ACA TTC AT-3′, R: 5′-ATG TGA GAA AAC TGA GAC AC -3′ (*miR-181a*). For single-point mutations, we used an Intron Muta-direct kit (Intron, Seoul, Korea). The sequences of these three vectors were confirmed by direct sequencing, and the SNPs were the only differences detected. To generate the miRNAs target gene::luciferase reporter constructs, similar to the cloning vectors, fragments of the *PAI-1* gene corresponding to the 3′-UTR region clone (OriGene, Rockville, MD, USA) were amplified and cloned into the pGL4.13-luciferase vector (Promega, Madison, WI, USA). The resulting cDNAs were PCR amplified using the following primers: forward 5′-CCC TGG GGA AAG ACG CCT T-3′ and reverse 5′-TTC GTA TTT ATT TAT TTT ATT TTT T-3′ with *Xba*I (TCTAGA)and *Fse*I (GGCCGGCC) linker (New England Biolabs, Ipswich, MA, USA), and all constructs were verified by sequencing. Cells from a human endometrial cell line (Ishikawa) were plated at 1 × 10^6^ cells per well in 6-well plates and transfected 24 h later using JetPRIME transfection reagent (Polyplus, France). Transfection reactions for miR-10a contained 500 ng of miR10a-A (in pcDNA3.1-) or 500 ng of miR-10a-T (in pcDNA3.1-) with 500 ng of 3′-UTR-PAI-1 in pGL4.13 and 200 ng of pGL4.75 (Renilla-normalization control); for miR-30c, reactions contained 500 ng of miR-30c-A (in pcDNA3.1-) or 500 ng of miR-30c-G (in pcDNA3.1-) with 500 ng of 3′-UTR-PAI-1 in pGL4.13 and 200 ng of pGL4.75 (Renilla-normalization control), for miR-181a2, reactions contained 500 ng of miR-181a-T (in pcDNA3.1-) or 500 ng of miR-181a-G (in pcDNA3.1-) with 500 ng of 3′-UTR-PAI-1 in pGL4.13 and 200 ng of pGL4.75 (Renilla-normalization control).

### 2.8. Quantitative Real-Time PCR (miR-10a, miR-30c, miR-181a Pre- and Mature-Form Primers)

TRIzol reagent (Invitrogen, Waltham, MA, USA) was used to isolate total RNA from Ishikawa cells that were transfected with 2.5 μg of vector after 16 h. Total RNA was then reverse transcribed using an M-MLV reverse transcriptase PCR kit (Biofact, Co., Ltd., Daejeon, Korea) and random or oligo dT20 primers (Invitrogen, Waltham, Massachusetts, USA) in addition to specific primers for *PAI-1* and *glyceraldehyde 3-phosphate dehydrogenase* (*GAPDH)*. Quantitative real-time PCR (qPCR) was performed as 20 μL reactions, containing each sequence-specific primer and quantitative PCR master mix (Solgent, Co., Ltd., Daejeon, Korea), using a Rotor-Gene 6000 real-time PCR system (Qiagen, Co., Ltd., Hilden, Germany). Expression levels were calculated according to the comparative threshold cycle (Ct) method using the formula 2^−ΔΔCt^. Primer sequences for amplification were as follows: has-miR-10a-pre forward: 5′-CCG AAT TTG TGT AAG GAA TTT TG-3′ and reverse 5′-AAG AGC GGA GTG TTT ATG TCA A-3′; has-miR-10a-mature forward: 5′-TAC CCT GTAG ATC CGA ATT T and reverse: universal primer (Qiagen Cat# 218193); has-miR-30c-pre forward: 5′-TGT GTA AAC ATC CTA CAC TCT CAG C-3′ and reverse: 5′-CCA TGG CAG AAG GAG TAA ACA-3′; has-miR-30c-mature forward: 5′-AAA CAT CCT ACA CTC TCA GC-3′ and reverse universal primer (Qiagen Cat# 218193); has-*miR-181a*-pre forward:5′-TAT CAG GCC AGC CTT CAG AG-3′ and reverse: 5′-AAT CCC AAA CTC ACC GAC AG-3′; *miR-181a*-mature forward:5′- TTC AAC GCT GTC GGT GAG TT-3′ and reverse: universal primer (Qiagen Cat# 218193); Human RNU6B (RNU6-2) forward:5′-ACG CAA ATT CGT GAA GCG TT-3′ and reverse universal primer (Qiagen Cat# 218193).

### 2.9. Prediction of miRNA Binding and Luciferase Reporter Assay

An online search was conducted to identify targets for *miR-10a*, *miR-30c*, *miR-181a*, and *miR-499b* using the TargetScan (http://www.targetscan.org (accessed on 21 May 2018)) and miRIAD databases (http://bmi.ana.med.uni-muenchen.de/miriad/ (accessed on 16 May 2018)). We used these databases to predict miRNAs that target overlapping regions of *PAI-1* mRNA transcripts. Target mRNA sequences, particularly within the 3′-UTR, are often obtained from the National Center for Biotechnology Information (www.ncbi.nlm.nih.gov/ (accessed on 26 September 2018)). We found that *miR-30c, miR-10a*, and *miR-181a* were predicted to be targets of the *PAI-1* 3′-UTR. Therefore, a luciferase reporter assay was used to evaluate the roles of *miR-30c, miR-10a,* and *miR-181a* in regulating the expression of target genes, as previously described. Briefly, wild-type pGL4.13-luciferase vector (Promega, Madison, WI, USA). constructs containing the 3′-UTRs of the *PAI-1* gene were generated by amplifying the 3′-UTR region clone (OriGene, Rockville, MD, USA) and cloning the amplification products into the downstream region of the pGL4.13 vector (Promega, Madison, WI, USA) using the *Xba*I and *Fse*I endonucleases (New England BioLabs, Ipswich, MA, USA). Positive clones were selected by sequence-specific PCR, restriction enzyme digestion, and DNA sequencing. Ishikawa cells were cultured in Dulbecco’s modified Eagle’s medium (DMEM) (Thermo Fisher Scientific, Inc. Waltham, Massachusetts, USA). All medium was supplemented with 10% fetal bovine serum (FBS) (Thermo Fisher Scientific, Inc. Waltham, Massachusetts, USA) and 1% penicillin/streptomycin (Thermo Fisher Scientific, Inc. Waltham, Massachusetts, USA). All cell lines were maintained in a CO_2_ incubator (5% CO_2_) at 37 °C. The Ishikawa cells used in this study were endometrial and are commonly used in RPL studies. Next, *miR-10a, miR-30c,* and *miR-181a* mimics (50 nM) were co-transfected into Ishikawa cells with 200 ng of the 3′-UTR of *PAI-1* in pGL4.13 constructs using lipofectamine 2000 (Invitrogen, Carlsbad, CA, USA). After 16 h of incubation, the luciferase activity was measured using a dual-luciferase reporter assay system (Promega, Madison, WI, USA). Each transfection was performed as triplicates.

## 3. Results

### 3.1. Baseline Characteristics of Recurrent Pregnancy Loss Patients and Control Subjects

The characteristics of RPL patients and control subjects are summarized in Table 1. The mean age was approximately 33 years for both groups, and both groups were 100% female. PLT count, aPTT, and concentrations of E2 and LH were greater in RPL patients than in controls (*p* = 0.0007, *p* = 0.005, *p* = 0.001, and *p* = 0.011, respectively). There were no significant differences in age, BMI, uric acid level, or FSH level between the two groups.

### 3.2. Genotype Frequencies of miRNA Polymorphisms According to the Number of Recurrent Pregnancy Losses

Table 2 shows the distribution of genotypes in RPL patients with ≥3 or ≥4 pregnancy losses and control subjects. Significant differences in the *miR-10a* SNP were observed between the RPL and control groups and were significantly correlated with RPL prevalence. Consistently, the absence of these miRNA polymorphisms showed a negative correlation with RPL. The associations of these polymorphisms were very interesting in RPL patients because the miRNA polymorphisms were related to decreased RPL, but they were not associated with RPL risk (Table 2). In addition, the number of RPL patients with risk factors was very small. Therefore, the associations with RPL occurrence will require further investigation. *miR-10a*A>T (chr17:48579816, rs3809783), *miR-30c*A>G (chr6:71377017, rs113749278), *miR-181a*T>C (chr9:124692981, rs16927589), and *miR-499b*A>G (chr20: 34990400, rs37464444) were all in the miRNA mature-form (rs3746444, rs-formnp8978) or pri-form (rs3809783, rs16927589). The SNPs in miRNA genes, including pri-miRNAs, pre-miRNAs, and mature miRNAs, could potentially influence the processing and/or target selection of miRNAs. Since we selected four SNPs in pri-form or mature-form, we wanted to determine whether all these miRNAs could influence the expression and regulation of target genes. Based on the intensity of FAM and VIC fluorescence, samples were automatically classified into one of three groups corresponding to genotypes AA, AT, or TT of *miR-10a*A>T; AA, AT, or GG of *miR-30c*A>G; TT, TC, or CC of *miR-181a*T>C; and AA, AG, or GG of *miR-499b*A>G.

### 3.3. Adjusted Odds Ratios for Risk of Recurrent Pregnancy Loss Associated with miRNA Polymorphisms Combined with Clinical Factors

The *miR-30c*AG+GG genotype was associated with decreased risk of RPL for age < 33 years (odds ratio [OR] = 0.583; 95% confidence interval [CI] = 0.371–0.918; *p* = 0.022) (Table 3). However, the *miR-181a*TC+CC genotype was associated with increased risk of RPL for age < 33 years (OR = 1.677; 95% CI = 1.038–2.709; *p* = 0.035), and the *miR-499b*AG + GG genotype was associated with increased risk of RPL for age ≥ 33 years (OR = 1.631; 95% CI = 1.028–2.588; *p* = 0.038). The *miR-10a*AT+TT genotype was associated with increased risk of RPL for BMI ≥ 25 kg/m^2^ (OR = 2.840; 95% CI = 1.544–5.223; *p* = 0.001). The *miR-499b*AG + GG genotype was associated with increased risk of RPL for BMI <25 kg/m^2^ (OR = 1.456; 95% CI = 1.029–2.059; *p* = 0.034) and with increased risk of RPL for BMI ≥25 kg/m^2^ (OR = 2.284; 95% CI = 1.377–3.789; *p* = 0.001). The *miR-181a*TC + CC genotype was associated with increased risk of RPL for PLT count <255.62×10^3^/μL) (OR = 1.779; 95% CI = 1.038–3.048; *p* = 0.036). Finally, the *miR-30c*AG+GG was associated with decreased risk of RPL for aPTT < 32.83 s (OR = 0.364; 95% CI = 0.185–0.717; *p* = 0.004).

### 3.4. Combination Analysis of miRNA Polymorphisms between Recurrent Pregnancy Loss Patients and Control Subjects

The results of combined gene-genotype analyses are shown in Table 4. The *miR-10a/miR-30c* combined genotype AT/AG was associated with increased RPL risk (OR = 2.156; 95% CI = 1.120–4.151; *p* = 0.022). The *miR-10a*A>T/*miR-181a*T>C combined genotype AT/TT was associated with increased RPL risk (OR = 1.974; 95% CI = 1.065–3.658; *p* = 0.031). The *miR-10aA>T/miR-499*A>G combined genotype AT/AG was associated with increased RPL risk (OR = 2.195; 95% CI = 1.156–4.169; *p* = 0.016). The *miR-30c*A>G*/miR-181a*T>C combined genotype AG/TT was also associated with increased RPL risk (OR = 1.839; 95% CI = 1.054–3.210; *p* = 0.032). Similarly, increased RPL risk was associated with the *miR-30c*A>G*/miR-499*A>G combined genotypes AA/GG (OR = 4.324; 95% CI = 1.423–13.141; *p* = 0.010) and AG/AG (OR = 1.921; 95% CI = 1.145–3.224; *p* = 0.013). The *miR-181a*T>C/*miR-499*A>G combined genotype TT/GG was also associated with increased RPL risk (OR = 8.320; 95% CI = 1.043–66.384; *p* = 0.046). However, after false-discovery rate (FDR)-*p* correction, there were no significant differences between RPL patients and controls in the ORs for the combined genotypes, except for the *miR-30c*A>G/*miR-499*A>G combined genotypes AA/GG and AG/AG.

### 3.5. Allele Combination Analysis of miRNA Polymorphisms in Recurrent Pregnancy Loss Patients and Control Subjects

The results of allele combination analyses of miRNA polymorphisms in RPL patients and control subjects are shown in Table 5 and Supplementary Tables S2–S4. The allele combinations *miR-10a/miR-30c/miR-181a/miR-499b* A-T-G-G (OR = 1.952; 95% CI = 1.120–3.149; *p* = 0.006), A-C-A-G (OR = 2.343; 95% CI = 1.111–4.942; *p* = 0.026), A-C-G-A (OR = 2.136; 95% CI = 1.095–4.165; *p* = 0.028), T-T-G-A (OR = 0.455; 95% CI = 0.215–0.962; *p* = 0.044), and T-C-G-A (OR = 13.020; 95% CI = 0.739–229.300; *p* = 0.017) were associated with an increased risk of RPL. However, after FDR-*p* correction, there were no significant differences between RPL patients and controls in the ORs of the allele combinations, except for the A-T-G-G and T-C-G-A allele combinations.

### 3.6. Differential Expression of the miR-10aA>T, miR-30cA>G, and miR-499bA>G Polymorphisms

**Figure 1:**
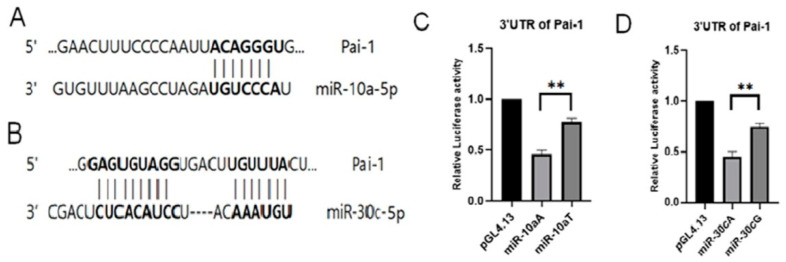
Expression of *miR-10a*A>T, *miR-30c*A>G, and the regulation of 3′-UTR of PAI-1 by *miR-10a* and *miR-30c*. (**A**,**B**) A schematic representation of gene with 3′-UTR of *PAI-1* that contain possible *miR-10a* and *miR-30c* binding sites in conserved regions. (**C**) Dual-luciferase reporter assays were performed to test the interaction of hsa-*miR-10a*A>T and its targeting sequence in the PAI-1 3′-UTR using constructs containing the predicted targeting sequence (pGL4.13-PAI-1 3′-UTR) cloned into the 3′-UTR of the reporter gene. Luciferase expression levels were normalized against Renilla luciferase expression. Data represent three independent exper-iments with triplicate measurements. ** *p* < 0.05. (**D**) Dual-luciferase reporter assays were performed to test the interaction of *miR-30c*A>G and its target sequence in the PAI-1 3′-UTR using constructs containing the predicted targeting sequence (pGL4.13-PAI-1 3′-UTR) cloned into the 3′-UTR of the reporter gene. Luciferase expression levels were normalized against Renilla luciferase expression. Data represent three independent experiments with triplicate measurements. ** *p* < 0.05.

The impact of SNPs on the interaction of *miR-10a*A>T, *miR-30c*A>G, *miR-181a*T>C, and *miR-499b*A>G on their targets was investigated by constructing various expression plasmids (*pri-miR-10aA*, *pri-miR-10aG*, *pri-miR-30cA*, *pri-miR-30cG*, *pre-miR-181a*T, *pre-miR-181a*G, *pri-miR-499b*A, and *pri-miR-499b*G) under control of the cytomegalovirus (CMV) promoter with either the major or minor allele. These plasmids were used in a dual luciferase assay performed with the 3′UTR of PAI-1, one of the predicted targets of miR-10a, miR-30c and miR181a, in Ishikawa human endometrial cells. A schematic diagram of a gene with a 3′-UTR of PAI-1 containing possible *miR-10a* and *miR-30c* binding sites in a conserved region is shown in Figure 1A,B. The luciferase activity of the 3′UTR of PAI-1 was significantly lower in *pre-miR-10a* having the A allele as compare to *pre-miR-10a* having the T allele (*p* < 0.05) (Figure 1C). Similarly, the luciferase activity of the 3′UTR of PAI-1 was significantly lower in the pre-miR-30c with the A allele as compared to pre-miR-30c with the G allele (*p* < 0.05) (Figure 1D).

### 3.7. Differences of Various Clinical Parameters According to miRNA Polymorphisms in RPL Patients

**Figure 2:**
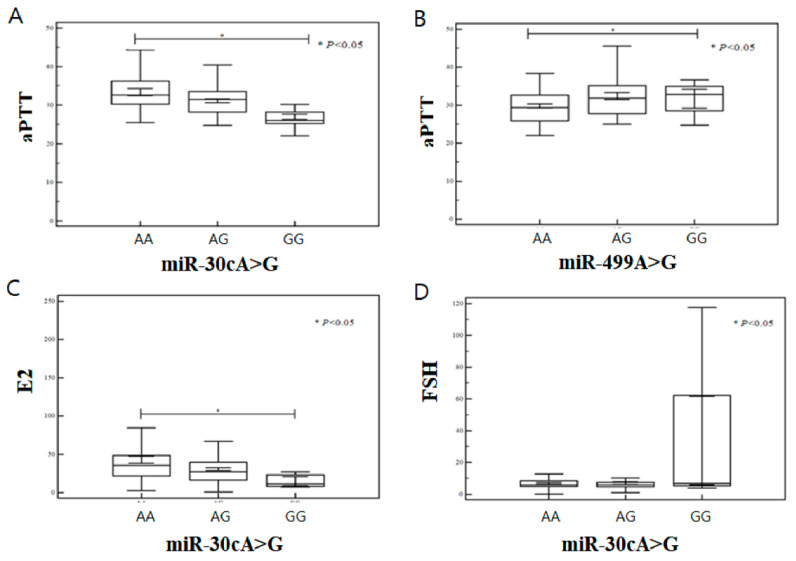
Analysis of variance of aPTT levels according to miRNA polymorphisms (PL ≥ 3) and analysis of variance of E2 and FSH levels according to miRNA polymorphisms. (**A**) The aPTT level was significantly different (*p* = 0.001) between miR-30cA>G AA (mean ± SD, 32.46 ± 4.71), AG (31.92 ± 4.11), and GG (27.56 ± 3.59). (**B**) The aPTT level was significantly different (*p* = 0.026) between miR-499 AA (mean ± SD, 31.20 ± 4.29), AG (32.62 ± 4.69), and GG (32.10 ± 4.18). (**C**) The E2 level was significantly different (*p* < 0.05) between miR-30cA>G AA (mean ± SD, 43.02 ± 38.58), AG (30.80 ± 19.30), and GG (15.10 ± 10.66). (**D**) The FSH level was significantly different (*p* < 0.05) between miR-30A>G AA (mean ± SD, 6.96 ± 4.29), AG (6.98 ± 8.47), and GG (33.82 ± 55.85).

Associations between miRNA polymorphisms and the levels of homocysteine, folate, total cholesterol, uric acid, blood urea nitrogen (BUN), estradiol (E2), thyroid-stimulating hormone (TSH), FSH, LH, prolactin, creatinine, platelets (PLT), as well as CD3^+^, CD4^+^, CD8^+^, CD19^+^, and CD56^+^ NK cells, in addition to the PT and aPTT were assessed by ordinal logistic regression analyses. We divided the risk factors into 10 grades and performed ordinal logistic regression using a proportional odds model. We found that the genotype frequency of *miR-30c*A>G was significantly associated with aPTT (AA: 32.46 ± 4.71, GG: 27.56 ± 3.59, *p* = 0.001), creatinine (AA: 1.19 ± 1.94, GG: 6.26 ± 3.71, *p* = 0.001), and E2 (AA: 1.19 ± 1.94, GG: 6.26 ± 3.71, *p* = 0.001). Levels of FSH differed significantly (*p* < 0.05) between the *miR-30c*A>G AA (mean ± SD, 32.36 ± 4.30 and 6.96 ± 4.29, respectively) and GG genotypes (30.49 ± 3.02 and 33.82 ± 55.85, respectively) (Table 6, Figure 2A,C,D). Additionally, levels of hematocrit (Hct) and total cholesterol (T. chol) differed significantly (*p* < 0.05) between the *miR-30c*AA and GG genotypes (36.65 ± 3.73 and 34.14 ± 4.49, 172.56 ± 64.85 and 38.15 ± 76.11, respectively). The *miR-181a*T>C genotype frequency was significantly associated with levels of creatinine (TT: 2.38±3.24, TC: 1.17±1.76, *p* = 0.011), Hcy (TT: 6.76 ± 2.01, CC: 9.98 ± 4.50, *p* = 0.001), LH (TT: 4.81 ± 2.74, CC: 4.20 ± 0.71, *p* = 0.038), PT (TT: 11.43 ± 1.14, CC: 10.20 ± 0.28, *p* = 0.048), and T. chol (TT: 136.96 ± 86.18, TC: 185.65 ± 76.23, *p* = 0.001). The *miR-499b*A>G genotype frequency was significantly associated with aPTT (TT: 31.20 ± 4.29, GG: 32.10 ± 4.18, *p* = 0.026) (Table 6, Figure 2B).

## 4. Discussion

Increasing evidence suggests that miRNAs play critical roles in the pathophysiology of various reproductive disorders [14,15,32]. Here, we investigated whether four pre-miRNA SNPs (*miR*-*10a, miR-30c, miR-181a*, and *miR-499b*) were associated with the risk of RPL in a cohort of Korean women. Specifically, we focused on the genotypes and allele combination of the selected miRNA polymorphisms and aimed to determine how they affected the risk of RPL. Using a genotype-based analysis method, we found that the GG and dominant (AA vs. AG + GG) *miR-499b* genotypes were significantly more common in RPL patients (PL ≥ 3 and PL ≥ 4, *p* < 0.05) than control subjects. In allele combination analyses, the AA/GG and AG/AG genotypes of *miR-30c*A>G/*miR-499*A>G were significantly more common in RPL patients than in controls.

As the activities of many genes are interconnected in complex conditions such as RPL, gene-gene interactions may affect gene-disease associations. The MDR method enables the detection of gene-gene interactions, regardless of the chromosomal locations of the genes [33]. We used a novel genotype-based MDR approach to examine the effects of potential interactions between different miRNAs on RPL risk. These results of these analyses, which examined the effects of four miRNA polymorphisms associated with RPL, suggested that gene-gene interactions involving these four miRNA polymorphisms also play roles in determining the risk of RPL. Allele combination MDR analyses indicated that the two combination conferred by *the miR-10a*A>T/*miR-181a*T>C/*miR-30c*A>G/*miR-499*A>G (A-T-G-G and T-C-G-A), the two combination conferred by the *miR-10a*A>T/*miR-181a*T>C/*miR-30c*A>G (T-T-A, T-C-G), the two combination conferred by the *miR-10a*A>T/*miR-30c*A>G/*miR-499*A>G allele combination (C-A-G, C-G-A), and the genotype conferred by the *miR-10a*A>T/*miR-30c*A>G allele combination (T-A) occur more frequently in patients with RPL than control subjects, suggesting a significant association with increased risk of RPL (all *p* < 0.05). In addition, the *miR-10a*A>T/*miR-181a*T>C/*miR-30c*A>G allele combination T-T-G and the *miR-10a*A>T/*miR-30c*A>G/*miR-499*A allele combination C-G-G were found to be less frequent in RPL patients than controls, suggesting these combinations exert a protective effect (all *p* < 0.05).

SNPs that occur in miRNA genes, miRNA machinery genes, or miRNAs that target genes involved in miRNA synthesis or function could adversely affect downstream gene expression [34]. Several studies have provided evidence supporting the critical role of miRNAs in RPL [35]. A previous study demonstrated that *miR-499* was associated with the transforming growth factor (TGF)-β signaling pathway [24]. Furthermore, the 3′-UTR of the *TGF-β3* gene has been shown to contain a putative binding site for *miR-30c* (rs928508) (http://www.targetscan.org (accessed on 21 May 2018)), which targets the drug metabolism gene *SULT1A1* [25]. Several TGF-β superfamily members perform critical functions in the female reproductive system. Specifically, these proteins regulate all processes of ovarian follicle development, including granulosa and theca cell proliferation, primordial follicle recruitment, gonadotropin receptor expression, ovulation, oocyte maturation, luteinization, and corpus luteum formation [36]. Additionally, the 3′-UTR of the prostaglandin F2 receptor inhibitor gene has been shown to contain a predicted binding target for *miR-604* (http://www.targetscan.org (accessed on 21 May 2018)), and prostaglandin F2 is required for placenta retention [37]. Furthermore, the *miR-10a*A>T polymorphism has been associated with regulation of IL-6 expression [26], and a previous study reported abnormal IL-6 expression in both animal models and patients with recurrent spontaneous abortions [38].

An online search for *miR-10a, miR-30c, miR-181a,* and *miR-499b* targets using the Target Scan and miRIAD databases (http://bmi.ana.med.uni-muenchen.de/miriad/ (accessed on 21 May 2018)) returned many putative mRNA targets. Among these targets, we focused on *PAI-1* for further functional analyses of *miR-10a, miR-30c,* and *miR-181a* because this gene has been shown to play several important roles in pregnancy and infertility [27]. PAI-1 is the primary inhibitor of plasminogen activators, including tPA and uPA. In the human placenta, *PAI-1* is expressed in the extravillous interstitial and vascular trophoblasts. During implantation and placentation, PAI-1 inhibits extracellular matrix degradation, which thereby inhibits trophoblast invasion. We reviewed the literature regarding various reproductive diseases in which PAI-1 plays a role. Elevated *PAI-1* levels have been detected in patients with RPL, preeclampsia, intrauterine growth restriction, gestational diabetes mellitus (GDM), endometriosis, and PCOS. Furthermore, both GDM and PCOS development have been reported to be related to the genetic role of the 4G/5G polymorphism in *PAI-1. In general, elevated blood levels of PAI-1* are associated with an increased risk of infertility and poor pregnancy outcomes. In contrast, deficiency of *PAI-1* results in transiently impaired placentation in mice [39], and deficiency of the *PAI-1* gene is associated with abnormal bleeding after trauma or surgery in humans [40]. PAI-1 functions as a major inhibitor of fibrinolysis, and its overexpression leads to fibrin accumulation and placental insufficiency during pregnancy. PAI-1 acts as a major inhibitor of fibrinolysis, resulting in fibrin accumulation and insufficient placental formation due to overexpression. Previous reports also suggested that elevation of PAI-1 levels is the most frequent hemostasis-related abnormality associated with unexplained RPL [41]. Thus, increased expression of PAI-1 leading to inhibition of fibrinolysis is believed to be the main cause of RPL.

To determine whether polymorphisms in *miR-10a, miR-30c,* and *miR-181a* affect target gene expression, we compared the expression levels of the 3′-UTR of *PAI-1* harboring the different polymorphisms of *miRNAs* in Ishikawa human endometrial cells. Aberrant *PAI-1* expression resulting from the expression of *miR-10a* with the A allele was significantly lower (*p* < 0.05) than aberrant *PAI-1* expression resulting from the expression of *miR-10a* with the T allele. In addition, the expression of *miR-30c* with the A allele was significantly lower (*p* < 0.05) than expression of premature and mature *miR-30c* with the G allele.

Expression of genotypes of *miR-30c*G as well as those of *miR-10a*T led to reduced expression of *PAI-1* mRNA. These results suggest that SNPs in *miR-30c* and *miR-10a* regulate the expression of the *PAI-1* gene. PAI-1-mediated inhibition of fibrinolysis and fibrin accumulation is currently believed to be the principal culprits for RPL; however, further studies are required to fully elucidate the underlying mechanisms.

FSH is the primary gonadotropin responsible for regulating the progression of pregnancy [42]. Optimal levels of FSH, especially during the first few months of pregnancy, are critical for proper formation of the placenta [43]. Our clinical data indicated significant changes in FSH levels in RPL patients harboring the *miR-30c*A>G polymorphism. We, therefore, hypothesized that abnormal regulation of *PAI-1* expression mediated by mutant *miR-30c* SNP results in aberrant FSH expression or disruption of the normal response to FSH. Imbalances in homocysteine and folate levels in particular are thought to contribute to low birth weight [44]. Specifically, higher homocysteine and lower folate concentrations during early pregnancy have been reported to be associated with lower placental weight and birth weight. However, we did not observe any associations between folate and homocysteine concentrations and placental weight.

We found that the dominant *miR-499b* AG genotype (AA vs. AG + GG) was significantly more frequent in RPL patients (*p* < 0.05). Earlier studies used a global approach to identify and profile miRNA expression at important stages during the estrous cycle and found a role of miRNAs in ovulation. Additionally, one-way ANOVA analysis of variance of data from RPL patients (Table 6) revealed that in comparison with *miR-30c*AA, the *miR-30c*GG genotype was associated with significantly lower aPTT, E2 (pg/mL), Hct, and T. chol (mg/dL) and significantly higher creatinine (mg/dL) and FSH (mIU/mL). Compared with *miR-181a*TT, the *miR-181a*CC genotype was associated with significantly higher homocysteine levels, suggesting this genotype is associated with increased risk of RPL (*p* < 0.05). Compared with *miR-181a*TT, the *miR-181a*TC genotype was associated with significantly higher T. chol levels, suggesting this genotype is associated with increased risk of RPL (*p* < 0.05). However, in the case of creatinine levels, the *miR-181a*TC genotype was associated with significantly lower levels than the *miR-181a*TT genotype, indicating a protective effect, although the results were inconsistent with OR and therefore, the difference was not significant.

## 5. Conclusions

We investigated the relationship between various miRNA polymorphisms and the occurrence and risk of RPL. Several genotypes and allele combinations were positively correlated with RPL occurrence and unfavorable prognoses according to reproductive disease risk factors, including FSH, LH, and E2 levels. However, this study has several limitations. First, how the miRNA polymorphisms in the *PAI-1* gene affect the development of RPL remains unclear. In addition to studies of *PAI-1*, future follow-up studies of other RPL-related genes and the *miR-10a* and *miR-30c* targets are planned, particularly studies of the role of genes related to the TGF-β signaling pathway. As TGF-β regulates cell proliferation, apoptosis, and homeostasis, it plays a critical role in regulating the progression of pregnancy. Second, the control subjects in our study were not completely healthy because some of them had sought medical attention for other issues. Our experience shows that recruiting healthy participants through imaging and laboratory testing results in significantly reduced enrollment rates. However, enrollment of participants without imaging and laboratory testing can introduce another challenge to risk factor assessment. Lastly, the study population was restricted to Korean patients. Although the results of our study provide the first evidence suggesting that miRNA polymorphisms in the *PAI-1* gene may serve as diagnostic and prognostic biomarkers for RPL, a prospective study involving a larger cohort of patients is warranted to validate these findings. A genome-wide analysis (using transcriptome-seq and miRNA-seq) is needed to identify the primary target genes, particularly the common genes regulated by these miRNAs. Determining the expression of these genes in the relevant gene-miRNA networks would provide stronger evidence in support of the results of the present research.

## Figures and Tables

**Table 1:** Baseline characteristics of recurrent pregnancy loss patients and control subjects.

Characteristics	Controls (*n* = 281)	RPL Patients (*n* = 381)	p *
Age (years, mean ± SD)	33.00 ± 5.73	33.03 ± 4.36	0.94
BMI (kg/m^2^, mean ± SD)	21.58 ± 3.18	21.35 ± 4.04	0.558
Previous pregnancy losses	None	3.01 ± 1.50	
Average no. of gestational weeks	39.28 ± 1.67	7.36 ± 1.93	<0.0001
CD56 NK cells (%, mean ± SD)	None	18.12 ± 7.98	
Homocysteine (μmol/L, mean ± SD)	None	6.98 ± 2.10	
Folate (nmol/L, mean ± SD)	None	14.18 ± 12.01	
Total cholesterol (mg/dL, mean ± SD)	None	187.73 ± 49.41	
Uric acid (mg/dL, mean ± SD)	4.19 ± 1.44	3.80 ± 0.83	0.172
PLT (10^3^/μL, mean ± SD)	235.17 ± 63.60	255.43 ± 59.22	0.0007
aPTT (sec, mean ± SD)	30.77 ± 4.60	32.23 ± 4.32	0.005
PAI-1 (ng/mL)	None	10.53 ± 5.72	
BUN (mg/dL)	None	9.98 ± 2.76	
Creatinine (mg/dL)	None	0.72 ± 0.12	
FSH (mIU/mL)	8.11 ± 2.84	7.51 ± 10.54	0.557
LH (mIU/mL)	3.32 ± 1.74	6.32 ± 12.11	0.011
E2 (pg/mL)	26.00 ± 14.74	35.64 ± 29.53	0.001
PT (sec, mean ± SD)	11.53 ± 3.10	11.58 ± 0.85	0.84

Abbreviations: aPTT, activated partial thromboplastin time; BMI, body mass index; BUN, blood. urea nitrogen; E2, estradiol; FSH, follicle-stimulating hormone; LH, luteinizing hormone; PLT, platelets; PT, prothrombin time; RPL, recurrent pregnancy loss; SD, standard deviation. * *p*-values were calculated by a two-sided t-test for continuous variables and a chi-square test for categorical variables.

**Table 2:** Genotype frequencies of miRNA gene polymorphisms in control subjects and recurrent pregnancy loss patients.

Genotype	Controls (*n* = 281)	RPL Patients (*n* = 381)	AOR (95% CI)	*p*	FDR-*p*	PL ≥ 3	AOR (95% CI)	*p*	FDR-*p*	PL ≥ 4	AOR (95% CI)	*p*	FDR-*p*
						(*n* = 201)				(*n* = 81)			
*miR-10a*A>T													
AA	230 (81.9)	285 (74.8)	1.000 (reference)			151 (75.1)	1.000 (reference)			60 (74.1)	1.000 (reference)		
AT	50 (17.8)	88 (23.1)	1.420(0.963–2.094)	0.077	0.584	44 (21.9)	1.365 (0.865–2.154)	0.181	0.362	19 (23.5)	1.470 (0.805–2.683)	0.21	0.21
TT	1 (0.4)	8 (2.1)	6.476(0.804–52.176)	0.079	0.237	6 (3.0)	9.484 (1.128–79.759)	0.038	0.274	2 (2.5)	7.931 (0.705–89.228)	0.094	0.156
Dominant (AA vs. AT+TT)			1.520(1.038–2.227)	0.032	0.709		1.524 (0.979–2.372)	0.062	0.124		1.595 (0.889–2.862)	0.117	0.156
Recessive (AA+AT vs. TT)			6.003(0.746–48.285)	0.092	0.276		8.847 (1.055–74.186)	0.045	0.631		7.206 (0.644–80.641)	0.109	0.156
HWE *P*	0.318	0.695				0.217				0.738			
*miR-30c*A>G													
AA	106 (37.7)	163 (42.8)	1.000 (reference)			130 (64.7)	1.000 (reference)			52 (64.2)	1.000 (reference)		
AG	144 (51.2)	182 (47.8)	0.821(0.591–1.139)	0.237	0.237	64 (31.8)	1.044 (0.647–1.686)	0.86	0.994	27 (33.3)	1.170 (0.640–2.139)	0.611	0.994
GG	32 (11.0)	36 (9.4)	0.742(0.432–1.276)	0.281	0.422	7 (3.5)	-	0.994	0.994	2 (2.5)	-	0.994	0.994
Dominant (AA vs. AG+GG)			0.810(0.591–1.111)	0.191	0.191		1.162 (0.724–1.864)	0.534	0.994		1.262 (0.696–2.288)	0.443	0.994
Recessive (AA+AG vs. GG)			0.842(0.506–1.400)	0.507	0.606		-	0.994	0.994		-	0.994	0.994
HWE *P*	0.104	0.144											
*miR-181a*T>C													
TT	198 (70.5)	247 (64.8)	1.000 (reference)			79 (39.3)	1.000 (reference)			32 (39.5)	1.000 (reference)		
TC	78 (27.8)	125 (32.8)	1.286(0.916–1.805)	0.147	0.221	104 (51.7)	1.376 (0.866–2.185)	0.177	0.223	42 (51.9)	1.403 (0.779–2.525)	0.259	0.345
CC	5 (1.8)	9 (2.4)	1.483(0.488–4.509)	0.487	0.487	18 (9.0)	2.094 (0.812–5.399)	0.126	0.223	7 (8.6)	2.035 (0.621–6.665)	0.241	0.345
Dominant (TT vs. TC+CC)			1.294(0.929–1.803)	0.128	0.191		1.448 (0.925–2.269)	0.106	0.223		1.462 (0.826–2.585)	0.192	0.345
Recessive (TT+TC vs. CC)			1.337(0.443–4.037)	0.606	0.606		1.764 (0.707–4.400)	0.223	0.223		1.664 (0.548–5.057)	0.369	0.369
HWE *P*	0.393	0.137											
*miR-499b*A>G													
AA	188 (66.9)	221 (58.0)	1.000 (reference)			116 (57.7)	1.000 (reference)			46 (56.8)	1.000 (reference)		
AG	87 (31.0)	139 (36.5)	1.361(0.977–1.896)	0.068	0.204	77 (38.3)	2.037 (1.240–3.347)	0.005	0.01	34 (42.0)	2.274 (1.241–4.168)	0.008	0.016
GG	6 (2.1)	21 (5.5)	2.956(1.168–7.482)	0.022	0.066	8 (4.0)	3.890 (0.767–19.730)	0.101	0.135	1 (1.2)	1.970 (0.152–25.590)	0.604	0.805
Dominant (AA vs. AG+GG)			1.465(1.062–2.020)	0.02	0.06		2.136 (1.314–3.472)	0.002	0.008		2.259 (1.240–4.114)	0.008	0.016
Recessive (AA+AG vs. GG)			2.677(1.066–6.725)	0.036	0.108		2.998 (0.605–14.857)	0.179	0.179		1.361 (0.111–16.739)	0.81	0.81
HWE *P*	0.263	0.888											

Abbreviations: AOR, adjusted odds ratio; CI, confidence interval; FDR-P, false-positive discovery rate-corrected; PL, pregnancy loss; RPL, recurrent pregnancy loss.

**Table 3:** Adjusted odds ratios for risk of recurrent pregnancy loss associated with miRNA polymorphisms combined with clinical factors.

Variable	*miR-10a*AT + TT		*miR-30c*AG + GG		*miR-181a*TC + CC		*miR-499b*AG + GG	
	AOR (95% CI)	*p*	AOR (95% CI)	*p*	AOR (95% CI)	*p*	AOR (95%CI)	*p*
Age (years)								
<33	1.476 (0.870–2.505)	0.149	0.583 (0.371–0.918)	0.02	1.677 (1.038–2.709)	0.035	1.329 (0.848–2.083)	0.216
≥33	1.566 (0.902–2.717)	0.111	1.124 (0.721–1.754)	0.606	0.996 (0.626–1.583)	0.985	1.631 (1.028–2.588)	0.038
Homocysteine								
<6.97µmol/L	1.186 (0.127–11.086)	0.881	0.364 (0.040–3.344)	0.372	-	-	3.063 (0.333–28.174)	0.323
≥6.97µmol/L	1.690 (0.190–15.041)	0.638	0.275 (0.032–2.399)	0.243	0.590 (0.124–2.816)	0.509	0.846 (0.177–4.050)	0.835
BMI								
<25 kg/m^2^	1.399 (0.928–2.108)	0.109	0.834 (0.593–1.174)	0.298	1.401 (0.979–2.005)	0.065	1.456 (1.029–2.059)	0.034
≥25 kg/m^2^	2.840 (1.544–5.223)	0.001	0.949 (0.591–1.524)	0.829	1.194 (0.725–1.967)	0.485	2.284 (1.377–3.789)	0.001
Platelet								
<255.62 × 10^3^/μL	1.133 (0.624–2.057)	0.681	1.008 (0.606–1.678)	0.976	1.779 (1.038–3.048)	0.036	1.468 (0.878–2.455)	0.144
≥255.62 × 10^3^/μL	2.019 (0.933–4.370)	0.075	0.539 (0.287–1.011)	0.054	0.820 (0.429–1.569)	0.55	1.256 (0.665–2.369)	0.483
PT								
≥11.58 s	1.476 (0.870–2.505)	0.149	1.845 (0.468–7.277)	0.382	0.557 (0.145–2.139)	0.394	0.368 (0.090–1.514)	0.166
<11.58 s	1.566 (0.902–2.717)	0.111	0.699 (0.335–1.458)	0.339	1.031 (0.495–2.151)	0.935	1.023 (0.522–2.006)	0.947
aPTT								
<32.83 s	1.476 (0.870–2.505)	0.149	0.364 (0.185–0.717)	0.004	1.714 (0.862–3.409)	0.125	1.409 (0.763–2.604)	0.273
≥32.83 s	1.566 (0.902–2.717)	0.111	0.976 (0.426–2.237)	0.954	1.069 (0.459–2.493)	0.877	0.639 (0.284–1.439)	0.279

Abbreviations: aPTT, activated partial thromboplastin time; AOR, adjusted odds ratio; BMI, body mass index; CI, confidence interval; PT, prothrombin time; RPL, recurrent pregnancy loss. The aPTT was below the 15% cut-off level in RPL patients and controls. Platelets were above the 15% cut-off level in RPL patients and controls.

**Table 4:** Combination analysis of miRNA polymorphisms between recurrent pregnancy loss patients and control subjects.

Genotype Combination	Controls (*n* = 281)	RPL Patients	AOR (95% CI)	*p* ^a^	FDR-*p* ^b^
		(*n* = 381)			
*miR-10a*A>T/*miR-30c*A>G					
AA/AA	162 (57.7)	190 (49.9)	1.000 (reference)		
AT/AA	35 (12.5)	51 (13.4)	1.243 (0.770–2.008)	0.374	0.499
AT/AG	14 (5.0)	35 (9.2)	2.156 (1.120–4.151)	0.022	0.088
AT/GG	1 (0.4)	2 (0.5)	1.770 (0.159–19.773)	0.643	0.643
TT/AA	1 (0.4)	6 (1.6)	4.958 (0.589–41.748)	0.141	0.282
*miR-10a*A>T/*miR-181a*T>C					
AA/TT	88 (31.3)	113 (29.7)	1.000 (reference)		
AA/TC	121 (43.1)	142 (37.3)	0.915 (0.632–1.324)	0.637	0.812
AA/CC	21 (7.5)	30 (7.9)	1.079 (0.576–2.020)	0.812	0.812
AT/TT	18 (6.4)	45 (11.8)	1.974 (1.065–3.658)	0.031	0.124
AT/TC	22 (7.8)	37 (9.7)	1.272 (0.698–2.317)	0.433	0.812
*miR-10a*A>T/*miR-499*A>G					
AA/AA	154 (54.8)	168 (44.1)	1.000 (reference)		
AA/AG	71 (25.3)	102 (26.8)	1.317 (0.907–1.914)	0.148	0.197
AA/GG	5 (1.8)	15 (3.9)	2.719 (0.964–7.665)	0.059	0.118
AT/AA	34 (12.1)	47 (12.3)	1.264 (0.772–2.069)	0.351	0.351
AT/AG	15 (5.3)	36 (9.4)	2.195 (1.156–4.169)	0.016	0.064
AT/GG	1 (0.4)	5 (1.3)	4.508 (0.520–39.109)	0.172	0.344
TT/AG	1 (0.4)	1 (0.3)	0.922 (0.057–14.874)	0.954	0.954
*miR-30c*A>G/*miR-181a*T>C					
AA/TT	79 (28.1)	101 (26.5)	1.000 (reference)		
AA/TC	89 (31.7)	120 (31.5)	1.058 (0.707–1.583)	0.784	0.784
AA/CC	30 (10.7)	26 (6.8)	0.692 (0.377–1.268)	0.233	0.466
AG/TT	25 (8.9)	58 (15.2)	1.839 (1.054–3.210)	0.032	0.128
AG/TC	52 (18.5)	59 (15.5)	0.886 (0.551–1.425)	0.617	0.784
*miR-30c*A>G/*miR-499*A>G					
AA/AA	137 (48.8)	146 (38.3)	1.000 (reference)		
AA/AG	57 (20.3)	83 (21.8)	1.355 (0.898–2.044)	0.147	0.196
AA/GG	4 (1.4)	18 (4.7)	4.324 (1.423–13.141)	0.01	0.026
AG/AA	49 (17.4)	68 (17.8)	1.319 (0.852–2.041)	0.214	0.214
AG/AG	27 (9.6)	55 (14.4)	1.921 (1.145–3.224)	0.013	0.026
AG/GG	2 (0.7)	2 (0.5)	0.908 (0.124–6.641)	0.924	0.924
*miR-181a*T>C/*miR-499*A>G					
TT/AA	72 (25.6)	98 (25.7)	1.000 (reference)		
TT/AG	33 (11.7)	54 (14.2)	1.155 (0.677–1.970)	0.597	0.796
TT/GG	1 (0.4)	11 (2.9)	8.320 (1.043–66.384)	0.046	0.184
TC/AA	95 (33.8)	107 (28.1)	0.818 (0.542–1.236)	0.34	0.68
TC/AG	47 (16.7)	67 (17.6)	1.040 (0.642–1.685)	0.872	0.872

Abbreviations: AOR, adjusted odds ratio; CI, confidence interval; RPL, recurrent pregnancy loss. ^a^ Fisher’s exact test. ^b^ False-discovery rate-adjusted *p*-value.

**Table 5:** Allele combination analysis of miRNA polymorphisms in recurrent pregnancy loss patients and control subjects.

Allele Combination	Controls	RPL Patients	OR (95% CI)	*p* ^a^	FDR-*p* ^b^
	(*n* = 281)	(*n* = 381)			
*miR-10a*A>T*/miR-181a*T>C*/miR-30c*A>G*/miR-499*A>G					
A-T-A-A	236 (41.9)	271 (35.6)	1.000 (reference)		
A-T-A-G	38 (6.9)	66 (8.6)	1.468 (0.953–2.263)	0.085	0.128
A-T-G-A	128 (22.9)	139 (18.2)	0.935 (0.695–1.257)	0.705	0.705
A-T-G-G	27 (4.9)	63 (8.2)	1.952 (1.210–3.149)	0.006	0.036
A-C-A-A	44 (7.8)	59 (7.7)	1.163 (0.759–1.784)	0.516	0.619
A-C-A-G	9 (1.7)	26 (3.5)	2.343 (1.111–4.942)	0.026	0.056
A-C-G-A	12 (2.3)	31 (4.1)	2.136 (1.095–4.165)	0.028	0.056
T-T-A-G	7 (1.2)	22 (3.0)	2.851 (1.202–6.764)	0.014	0.056
T-T-G-A	20 (3.7)	10 (1.4)	0.455 (0.215–0.962)	0.044	0.088
T-T-G-G	3 (0.7)	1 (0.2)	0.434 (0.079–2.391)	0.425	0.425
T-C-A-A	9 (1.6)	18 (2.3)	1.735 (0.765–3.937)	0.235	0.313
T-C-G-A	0 (0.0)	6 (0.9)	13.020 (0.739–229.300)	0.017	0.017

Abbreviations: CI, confidence interval; FDR, false-discovery rate; OR, odds ratio; RPL, recurrent pregnancy loss. ORs and 95% CIs for each allele combination were calculated with reference to frequencies of all others using Fisher’s exact test. ^a^ Fisher’s exact test. ^b^ FDR-adjusted *p*-value.

**Table 6:** Differences of various clinical parameters according to miRNA polymorphisms in RPL patients.

	aPTT	Creatinine (mg/dL)	E2 (pg/mL)	FSH (mIU/mL)	Hct	Hcy	LH (mIU/mL)	PT	T. Chol (mg/dL)
Genotype	Mean ± SD	Mean ± SD	Mean ± SD	Mean ± SD	Mean ± SD	Mean ± SD	Mean ± SD	Mean ± SD	Mean ± SD
*miR-10a* A>T									
AA	31.86 ± 4.43	1.87 ± 2.75	35.07 ± 25.54	7.84 ± 11.93	36.28 ± 3.90	6.92 ± 2.00	5.72 ± 7.14	11.61 ± 1.87	154.54 ± 83.14
AT	31.39 ± 4.64	2.47 ± 3.36	38.07 ± 41.91	6.30 ± 3.79	36.36 ± 4.20	7.31 ± 2.37	8.38 ± 21.83	11.41 ± 0.89	140.93 ± 89.67
TT	31.47 ± 3.42	0.73 ± 0.15	31.20 ± 9.11	9.94 ± 4.91	36.63 ± 6.47	5.64 ± 1.10	5.52 ± 1.60	11.80 ± 0.35	251.00 ± 118.01
*p*	0.719	0.53	0.837	0.642	0.974	0.228	0.435	0.696	0.079
*miR-30c* A>G									
AA	32.46 ± 4.71	1.19 ± 1.94	43.02 ± 38.58	6.96 ± 4.29	36.65 ± 3.73	6.84 ± 2.00	5.41 ± 3.63	11.83 ± 2.21	172.56 ± 64.85
AG	31.92 ± 4.11	1.55 ± 2.35	30.80 ± 19.30	6.98 ± 8.47	36.27 ± 4.02	7.01 ± 2.16	6.46 ± 14.43	11.41 ± 1.28	167.25 ± 79.17
GG	27.56 ± 3.59	6.26 ± 3.71	15.10 ± 10.66	33.82 ± 55.85	34.14 ± 4.49	7.73 ± 1.86	20.00 ± 32.76	11.68 ± 0.83	38.15 ± 76.11
*p*	0.001	0.001	0.015	0.001	0.013	0.199	0.062	0.135	0.001
*miR-181a* T>C									
TT	31.51 ± 4.45	2.38 ± 3.24	33.91 ± 21.97	6.42 ± 3.17	36.26 ± 4.08	6.76 ± 2.01	4.81 ± 2.74	11.43 ± 1.14	136.96 ± 86.18
TC	32.23 ± 4.48	1.17 ± 1.76	39.85 ± 41.63	9.76 ± 17.78	36.45 ± 3.70	7.36 ± 1.99	9.50 ± 20.64	11.91 ± 2.48	185.65 ± 76.23
CC	36.05 ± 3.32	-	25.67 ± 5.08	6.73 ± 1.80	31.35 ± 0.64	9.98 ± 4.50	4.20 ± 0.71	10.20 ± 0.28	-
*p*	0.17	0.011	0.409	0.115	0.19	0.001	0.038	0.048	0.001
*miR-499b*A>G									
AA	31.20 ± 4.29	2.14 ± 3.00	35.86 ± 26.02	6.84 ± 7.73	36.43 ± 3.85	7.00 ± 2.06	5.37 ± 4.90	11.54 ± 1.72	144.26 ± 86.46
AG	32.62 ± 4.69	1.82 ± 2.84	34.70 ± 37.33	9.32 ± 15.27	36.25 ± 4.24	7.01 ± 2.15	8.62 ± 20.48	11.54 ± 1.25	164.37 ± 84.75
GG	32.10 ± 4.18	1.67 ± 2.54	38.39 ± 23.85	5.46 ± 3.78	34.60 ± 3.54	6.80 ± 1.99	4.55 ± 4.01	12.17 ± 3.59	166.09 ± 82.36
*p*	0.026	0.66	0.94	0.251	0.18	0.942	0.198	0.453	0.223

Abbreviations: aPTT, activated partial thromboplastin time; E2, estradiol; FSH, follicle-stimulating hormone; Hct, hematocrit; T. chol, total cholesterol; Hcy, homocysteine level; LH, luteinizing hormone; RPL, recurrent pregnancy loss; PT, prothrombin time.

## Data Availability

The data that support the findings of this study are available from the corresponding author upon reasonable request.

## Supplementary Materials

The following supporting information can be downloaded at: https://www.mdpi.com/article/10.3390/biomedicines10102395/s1, Table S1: Genotype frequencies of miRNA gene polymorphisms in control subjects and recurrent pregnancy loss patients; Table S2: Four allele combination analysis of miRNA polymorphisms in recurrent pregnancy loss patients and control subjects; Table S3. Three allele combination analysis of miRNA polymorphisms in recurrent pregnancy loss patients and control subjects; Table S4. Two allele combination analysis of miRNA polymorphisms in recurrent pregnancy loss patients and control subjects.

## References

[1] Coulam C.B., Coulam C.B., Clark D.A., Beer A.E., Kutteh W.H., Kwak J., Stephenson M. (1997). Clinical Guidelines Recommendation Committee for Diagnosis and Treatment of Recurrent Spontaneous Abortion. Current clinical options for diagnosis and treatment of recurrent spontaneous abortion. Am. J. Reprod. Immunol..

[2] Rai R., Regan L. (2006). Recurrent miscarriage. Lancet.

[3] Sierra S., Stephenson M. (2006). Genetics of Recurrent Pregnancy Loss. Semin. Reprod. Med..

[4] Marjorie P.P., Perron M.P., Provost P. (2008). Protein interactions and complexes in human microRNA biogenesis and function. Front. Biosci..

[5] Bartel D.P. (2004). MicroRNAs: Genomics, biogenesis, mechanism, and function. Cell.

[6] Ambros V. (2004). The functions of animal microRNAs. Nature.

[7] Lim L.P., Lau N., Garrett-Engele P., Grimson A., Schelter J.M., Castle J., Bartel D.P., Linsley P.S., Johnson J.M. (2005). Microarray analysis shows that some microRNAs downregulate large numbers of target mRNAs. Nature.

[8] Cuellar T.L., McManus M.T. (2005). MicroRNAs and endocrine biology. J. Endocrinol..

[9] Lee Y., Kim M., Han J., Yeom K.-H., Lee S., Baek S.H., Kim V.N. (2004). MicroRNA genes are transcribed by RNA polymerase II. EMBO J..

[10] Gregory R.I., Chendrimada T.P., Shiekhattar R. (2006). MicroRNA Biogenesis: Isolation and Characterization of the Microprocessor Complex. MicroRNA Protoc..

[11] Yi R., Qin Y., Macara I.G., Cullen B.R. (2003). Exportin-5 mediates the nuclear export of pre-microRNAs and short Hairpin RNAs. Genes Dev..

[12] Moore M.S., Biobel G. (1993). The GTP-binding protein Ran/TC4 is required for protein import into the nucleus. Nature.

[13] O’Toole A.S., Miller S., Haines N., Zink M.C., Serra M.J. (2006). Comprehensive thermodynamic analysis of 3’ double-nucleotide overhangs neighboring Watson-Crick terminal base pairs. Nucleic Acids Res..

[14] Song F.J., Chen K.X. (2011). Single-nucleotide polymorphisms among microRNA: Big effects on cancer. Chin. J. Cancer.

[15] Imbar T., Eisenberg I. (2014). Regulatory role of microRNAs in ovarian function. Fertil. Steril..

[16] Iwai N., Naraba H. (2005). Polymorphisms in human pre-miRNAs. Biochem. Biophys. Res. Commun..

[17] Lal A., Navarro F., Maher C.A., Maliszewski L.E., Yan N., O’Day E., Chowdhury D., Dykxhoorn D.M., Tsai P., Hofmann O. (2009). miR-24 inhibits cell proliferation by targeting E2F2, MYC, and other cell-cycle genes via binding to “seedless” 3′UTR microRNA recognition elements. Mol. Cell.

[18] He X.-Y., Chen J.-X., Zhang Z., Li C.-L., Peng Q., Peng H.-M. (2010). The let-7a microRNA protects from growth of lung carcinoma by suppression of k-Ras and c-Myc in nude mice. J. Cancer Res. Clin. Oncol..

[19] Toloubeydokhti T., Bukulmez O., Chegini N. (2008). Potential Regulatory Functions of MicroRNAs in the Ovary. Semin. Reprod. Med..

[20] Medeiros L.A., Dennis L.M., Gill M.E., Houbaviy H., Markoulaki S., Fu D., White A.C., Kirak O., Sharp P.A., Page D.C. (2011). *Mir-290–295* deficiency in mice results in partially penetrant embryonic lethality and germ cell defects. Proc. Natl. Acad. Sci. USA.

[21] Butz H., Likó I., Czirják S., Igaz P., Korbonits M., Rácz K., Patócs A. (2011). MicroRNA profile indicates downregulation of the TGF pathway in sporadic non-functioning pituitary adenomas. Pituitary.

[22] Nguyen Dien G.T., Smith R.A., Haupt L.M., Griffiths L.R., Nguyen H.T. (2014). Genetic polymorphisms inmiRNAs targeting the estrogen receptor and their effect on breast cancer risk. Meta Gene.

[23] Santamaria X., Taylor H. (2014). MicroRNA and gynecological reproductive diseases. Fertil. Steril..

[24] Suzuki H.I. (2018). MicroRNA Control of TGF-β Signaling. Int. J. Mol. Sci..

[25] Knight P.G., Glister C. (2006). TGF-beta superfamily members and ovarian follicle development. Reproduction.

[26] Dong K., Xu Y., Yang Q., Shi J., Jiang J., Chen Y., Song C., Wang K. (2017). Associations of functional microRNA binding site polymorphisms in IL23/Th17 inflammatory pathway genes with gastric cancer risk. Mediat. Inflamm..

[27] Li C., Zhu H., Bai W., Su L.-L., Liu J.-Q., Cai W.-X., Zhao B., Gao J.-X., Han S.-C., Li J. (2015). MiR-10a and miR-181c regulate collagen type I generation in hypertrophic scars by targeting PAI-1 and uPA. FEBS Lett..

[28] Ryu C.S., Sakong J.H., Ahn E.H., Kim J.O., Ko D., Kim J.H., Lee W.S., Kim N.K. (2019). Association study of the three functional polymorphisms (TAS2R46G>A, OR4C16G>A, and OR4X1A>T) with recurrent pregnancy loss. Genes Genom..

[29] Wang H., Wu S., Wu J., Sun S., Wu S., Bao W. (2018). Association analysis of the SNP (rs345476947) in the FUT2 gene with the production and reproductive traits in pigs. Genes Genom..

[30] Hochberg Y., Benjamini Y. (1990). More powerful procedures for multiple significance testing. Stat. Med..

[31] Ritchie M.D., Hahn L.W., Roodi N., Bailey L.R., Dupont W.D., Parl F.F., Moore J.H. (2001). Multifactor-Dimensionality Reduction Reveals High-Order Interactions among Estrogen-Metabolism Genes in Sporadic Breast Cancer. Am. J. Hum. Genet..

[32] Teague E.M.C.O., Print C., Hull M.L. (2010). The role of microRNAs in endometriosis and associated reproductive conditions. Hum. Reprod. Updat..

[33] Hahn L.W., Ritchie M.D., Moore J.H. (2003). Multifactor dimensionality reduction software for detecting gene-gene and gene-environment interactions. Bioinformatics.

[34] Moszyńska A., Gebert M., Collawn J.F., Bartoszewski R. (2017). SNPs in microRNA target sites and their potential role in human disease. Open Biol..

[35] Barchitta M., Maugeri A., Quattrocchi A., Agrifoglio O., Agodi A. (2017). The Role of miRNAs as Biomarkers for Pregnancy Outcomes: A Comprehensive Review. J. Genom..

[36] Liu X., Li M., Peng Y., Hu X., Xu J., Zhu S., Yu Z., Han S. (2016). miR-30c regulates proliferation, apoptosis and differentiation via the Shh signal-ing pathway in P19 cells. Exp. Mol. Med..

[37] Bider D., Dulitzky M., Goldenberg M., Lipitz S., Mashiach S. (1996). Intraumbilical vein injection of prostaglandin F2α in retained placenta. Eur. J. Obstet. Gynecol. Reprod. Biol..

[38] Wolff M.V., Thaler C.J., Strowitzki T., Broome J., Stolz W., Tabibzadeh S. (2000). Regulated expression of cytokines in human endometrium throughout the menstrual cycle: Dysregulation in habitual abortion. Mol. Hum. Reprod..

[39] Labied S., Blacher S., Carmeliet P., Noël A., Frankenne F., Foidart J.-M., Munaut C. (2011). Transient reduction of placental angiogenesis in PAI-1-deficient mice. Physiol. Genom..

[40] Fay W.P., Parker A.C., Condrey L.R., Shapiro A.D. (1997). Human plasminogen activator inhibitor-1 (PAI-1) deficiency: Characterization of a large kindred with a null mutation in the PAI-1 gene. Blood.

[41] Gris J.-C., Ripart-Neveu S., Maugard C., Tailland M.L., Brun S., Courtieu C., Biron C., Hoffet M., Hédon B., Marès P. (1997). Respective evaluation of the prevalence of haemostasis abnormalities in unexplained primary early recurrent miscarriages. The Nimes Obstetricians and Haematologists (NOHA) Study. Thromb. Haemost..

[42] Kim Y.K., Wasser S.K., Fujimoto V.Y., Klein N.A., Moore D.E., Soules M.R. (1997). Utility of follicle stimulating hormone (FSH), luteinizing hormone (LH), oestradiol and FSH:LH ratio in predicting reproductive age in normal women. Hum. Reprod..

[43] Stilley J.A.W., Segaloff D.L. (2018). FSH Actions and Pregnancy: Looking Beyond Ovarian FSH Receptors. Endocrinology.

[44] Bergen N.E., Jaddoe V.W., Timmermans S., Hofman A., Lindemans J., Russcher H., Raat H., Steegers-Theunissen R.P., Steegers E.A. (2012). Homocysteine and folate concentrations in early pregnancy and the risk of adverse pregnancy outcomes: The Generation R Study. BJOG.

