# Supplementary material for: Genetic Variations miR-10aA>T, miR-30cA>G, miR-181aT>C, and miR-499bA>G and the Risk of Recurrent Pregnancy Loss in Korean Women
Source: Biomedicines. 2022 Sep 25;10(10):2395. doi: 10.3390/biomedicines10102395 (PMC9598437; doi:10.3390/biomedicines10102395)
Supplement: Supplementary file 1 [file biomedicines-10-02395-s001.zip › biomedicines-1755523-supplementary.pdf]

**Supplementary Table S1. Genotype frequencies of miRNA gene polymorphisms in control subjects and recurrent pregnancy loss patients.**

| Genotype | Controls (n=281) | RPL patients (n=381) | AOR (95% CI) | *P* | FDR-*P* |
|---|---|---|---|---|---|
| *miR-10a*A>T | | | | | |
| AA | 230 (81.9) | 285 (74.8) | 1.000 (reference) | | |
| AT | 50 (17.8) | 88 (23.1) | 1.420 (0.963–2.094) | 0.077 | 0.584 |
| TT | 1 (0.4) | 8 (2.1) | 6.476 (0.804–52.176) | 0.079 | 0.237 |
| Dominant (AA vs. AT+TT) | | | 1.520 (1.038–2.227) | **0.032** | 0.709 |
| Recessive (AA+AT vs. TT) | | | 6.003 (0.746–48.285) | 0.092 | 0.276 |
| HWE *P* | 0.318 | 0.695 | | | |
| *miR-30c*A>G | | | | | |
| AA | 106 (37.7) | 163 (42.8) | 1.000 (reference) | | |
| AG | 144 (51.2) | 182 (47.8) | 0.821 (0.591–1.139) | 0.237 | 0.237 |
| GG | 32 (11.0) | 36 (9.4) | 0.742 (0.432–1.276) | 0.281 | 0.422 |
| Dominant (AA vs. AG+GG) | | | 0.810 (0.591–1.111) | 0.191 | 0.191 |
| Recessive (AA+AG vs. GG) | | | 0.842 (0.506–1.400) | 0.507 | 0.606 |
| HWE-*p* | 0.104 | 0.144 | | | |
| *miR-181a*T>C | | | | | |
| TT | 198 (70.5) | 247 (64.8) | 1.000 (reference) | | |
| TC | 78 (27.8) | 125 (32.8) | 1.286 (0.916–1.805) | 0.147 | 0.221 |
| CC | 5 (1.8) | 9 (2.4) | 1.483 (0.488–4.509) | 0.487 | 0.487 |
| Dominant (TT vs. TC+CC) | | | 1.294 (0.929–1.803) | 0.128 | 0.191 |
| Recessive (TT+TC vs. CC) | | | 1.337 (0.443–4.037) | 0.606 | 0.606 |
| HWE-*p* | 0.393 | 0.137 | | | |
| *miR-499b*A>G | | | | | |
| AA | 188 (66.9) | 221 (58.0) | 1.000 (reference) | | |
| AG | 87 (31.0) | 139 (36.5) | 1.361 (0.977–1.896) | 0.068 | 0.204 |
| GG | 6 (2.1) | 21 (5.5) | 2.956 (1.168–7.482) | **0.022** | 0.066 |
| Dominant (AA vs. AG+GG) | | | 1.465 (1.062–2.020) | **0.020** | 0.060 |
| Recessive (AA+AG vs. GG) | | | 2.677 (1.066–6.725) | **0.036** | 0.108 |
| HWE-*p* | 0.263 | 0.888 | | | |

Abbreviations: AOR, adjusted odds ratio; CI, confidence interval; FDR-*P*, false-positive discovery rate-corrected; PL, pregnancy loss; RPL, recurrent pregnancy loss.

**Supplementary Table S2. Four allele combination analysis of miRNA polymorphisms in recurrent pregnancy loss patients and control subjects.**

| Allele combination | Control (n= 281) | Case (n=381) | OR(95% CI) | $P^{a}$ | FDR-*P* |
|---|---|---|---|---|---|
| ***miR-10a*A>T*/miR 181a* T>C/*miR-30c*A>G/*miR-499*A>G** | | | | | |
| A-T-A-A | 236 | 272 | 1.000 (reference) | | |
| A-T-A-G | 39 | 66 | 1.468 (0.953 - 2.263) | 0.085 | 0.128 |
| A-T-G-A | 129 | 139 | 0.935 (0.695 - 1.257) | 0.705 | 0.705 |
| A-T-G-G | 28 | 63 | 1.952 (1.210 - 3.149) | **0.006** | 0.036 |
| A-C-A-A | 44 | 59 | 1.163 (0.759 - 1.784) | 0.516 | 0.619 |
| A-C-A-G | 10 | 27 | 2.343 (1.111 - 4.942) | **0.026** | 0.056 |
| A-C-G-A | 13 | 32 | 2.136 (1.095 - 4.165) | **0.028** | 0.056 |
| A-C-G-G | 12 | 0 | 0.035 (0.002 - 0.590) | **0.0001** | 0.0001 |
| T-T-A-A | 11 | 44 | 3.471 (1.752 - 6.874) | **0.0001** | 0.0001 |
| T-T-A-G | 7 | 23 | 2.851 (1.202 - 6.764) | **0.014** | 0.056 |
| T-T-G-A | 21 | 11 | 0.455 (0.215 - 0.962) | **0.044** | 0.088 |
| T-T-G-G | 4 | 2 | 0.434 (0.079 - 2.391) | 0.425 | 0.425 |
| T-C-A-A | 9 | 18 | 1.735 (0.765 - 3.937) | 0.235 | 0.313 |
| T-C-A-G | 0 | 0 | - | - | |
| T-C-G-A | 0 | 7 | 13.020 (0.739 - 229.300) | **0.017** | 0.017 |
| T-C-G-G | 0 | 0 | - | - | |

Abbreviations: CI, confidence interval; FDR, false-discovery rate; OR, odds ratio; RPL, recurrent pregnancy loss. ORs and 95% CIs for each allele combination were calculated with reference to frequencies of all others using Fisher's exact test. [a]Fisher's exact test. [b]FDR-adjusted *P*-value.

**Supplementary Table S3. Three allele combination analysis of miRNA polymorphisms in recurrent pregnancy loss patients and control subjects.**

| Allele combination | Control (n= 281) | Case (n=381) | OR(95% CI) | $P^a$ | FDR-*P* |
|---|---|---|---|---|---|
| ***miR-10a*A>T/*miR-181a*T>C/*miR-30c*A>G** | | | | | |
| A-T-A | 275 | 340 | 1.000 (reference) | | |
| A-T-G | 156 | 201 | 1.042 (0.801 - 1.355) | 0.789 | 1.000 |
| A-C-A | 53 | 84 | 1.282 (0.878 - 1.872) | 0.216 | 0.432 |
| A-C-G | 26 | 33 | 1.027 (0.599 - 1.758) | 1.000 | 1.000 |
| T-T-A | 19 | 64 | 2.724 (1.594 - 4.658) | 0.0001 | 0.000 |
| T-T-G | 24 | 13 | 0.438 (0.219 - 0.877) | 0.026 | 0.039 |
| T-C-A | 9 | 20 | 1.797 (0.805 - 4.011) | 0.181 | 0.181 |
| T-C-G | 0 | 7 | 12.140 (0.690 - 213.600) | 0.020 | 0.039 |
| ***miR-181a*T>C/*miR-30c*A>G/*miR-499*A>G** | | | | | |
| T-A-A | 242 | 316 | 1.000 (reference) | | |
| T-A-G | 45 | 89 | 1.515 (1.020 - 2.250) | 0.041 | 0.090 |
| T-G-A | 154 | 150 | 0.746 (0.564 - 0.987) | 0.045 | 0.090 |
| T-G-G | 32 | 64 | 1.532 (0.971 - 2.417) | 0.073 | 0.097 |
| C-A-A | 60 | 77 | 0.983 (0.674 - 1.433) | 0.924 | 0.924 |
| C-A-G | 8 | 26 | 2.489 (1.107 - 5.596) | 0.031 | 0.031 |
| C-G-A | 7 | 38 | 4.157 (1.825 - 9.473) | 0.000 | |
| C-G-G | 13 | 3 | 0.177 (0.050 - 0.627) | 0.004 | 0.008 |

Abbreviations: CI, confidence interval; FDR, false-discovery rate; OR, odds ratio; RPL, recurrent pregnancy loss. ORs and 95% CIs for each allele combination were calculated with reference to frequencies of all others using Fisher's exact test. [a]Fisher's exact test. [b]FDR-adjusted *P*-value.

**Supplementary Table S4. Two allele combination analysis of miRNA polymorphisms in recurrent pregnancy loss patients and control subjects.**

| Allele combination | Control (n= 281) | Case (n=381) | OR(95% CI) | *P*[a] | FDR-*P* |
|---|---|---|---|---|---|
| ***miR-10a*A>T/*miR-181a*T>C** | | | | | |
| A-T | 430 | 541 | 1.000 (reference) | | |
| A-C | 80 | 117 | 1.162 (0.851 - 1.587) | 0.386 | 0.386 |
| T-T | 44 | 78 | 1.409 (0.953 - 2.083) | 0.099 | 0.149 |
| T-C | 8 | 26 | 2.583 (1.158 - 5.764) | **0.021** | 0.063 |
| ***miR-10a*A>T/*miR-30c*A>G** | | | | | |
| A-A | 329 | 424 | 1.000 (reference) | | |
| A-G | 181 | 234 | 1.003 (0.788 - 1.277) | 1.000 | 1.000 |
| T-A | 27 | 84 | 2.414 (1.529 - 3.813) | **0.0001** | 0.0001 |
| T-G | 25 | 20 | 0.621 (0.339 - 1.137) | 0.125 | 0.125 |
| ***miR-181a*T>C/*miR-30c*A>G** | | | | | |
| T-A | 289 | 405 | 1.000 (reference) | | |
| T-G | 185 | 214 | 0.825 (0.644 - 1.058) | 0.145 | 0.435 |
| C-A | 66 | 103 | 1.114 (0.790 - 1.571) | 0.601 | 0.601 |
| C-G | 22 | 40 | 1.297 (0.755 - 2.230) | 0.419 | 0.601 |

Abbreviations: CI, confidence interval; FDR, false-discovery rate; OR, odds ratio; RPL, recurrent pregnancy loss. ORs and 95% CIs for each allele combination were calculated with reference to frequencies of all others using Fisher's exact test. [a]Fisher's exact test. [b]FDR-adjusted *P*-value.
